# Reliable and Reusable All‐Solid‐State Contact‐Type Pre‐Lithiation Platform for High‐Performance All‐Solid‐State Batteries

**DOI:** 10.1002/adma.74035

**Published:** 2026-07-08

**Authors:** Yunho Lee, Yongjun Kwon, Juhyeong Noh, Seok Hun Kang, Kyubeen Kang, Junhyeok Choi, Young‐Gi Lee, Jaecheol Choi, Hyeong Min Jin, Yong Min Lee, Ju Young Kim

**Affiliations:** ^1^ Smart Materials Research Section Electronics and Telecommunications Research Institute (ETRI) Daejeon Republic of Korea; ^2^ Department of Organic Materials Engineering Chungnam National University Daejeon Republic of Korea; ^3^ Department of Chemical and Biomolecular Engineering Yonsei University Seoul Republic of Korea; ^4^ Department of Semiconductor and Advanced Device Engineering University of Science and Technology (UST) Daejeon Republic of Korea; ^5^ Department of Battery Engineering Yonsei University Seoul Republic of Korea

## Abstract

All‐solid‐state batteries represent a promising approach for achieving high energy density and enhanced safety by utilizing bipolar cell architectures and nonflammable solid electrolytes. However, lithium loss during initial cycling occurs primarily due to the limited electrochemical stability of solid electrolytes and the irreversibility of anode materials with high theoretical capacities, resulting in a lower achievable energy density than theoretically expected. Additionally, all‐solid‐state batteries are highly sensitive to interfacial phenomena, which makes addressing these challenges crucial for maximizing their electrochemical performance. In this study, we introduce a reliable and reusable contact‐type pre‐lithiation platform specifically designed for all‐solid‐state systems. This platform, consisting of solid electrolytes and electron‐conductive agents, exhibits balanced ionic and electronic transport characteristics, enabling uniform pre‐lithiation of all‐solid‐state electrodes through simple, reversible physical contact. Even under a low stack pressure of 8 MPa, pre‐lithiation can be effectively controlled by contact time and operating temperature, while the low stack pressure effectively suppresses the formation of highly resistive decomposition products at the interface. Consequently, the precisely pre‐lithiated anodes with optimized interfacial characteristics significantly enhance Coulombic efficiency during initial cycles and overall cycling performance, contributing to the realization of high‐performance all‐solid‐state batteries with genuinely high energy density.

## Graphical abstract

A reusable contact‐type pre‐lithiation platform enables uniform, precisely controllable pre‐lithiation at low stack pressure, suppressing interfacial degradation and thereby markedly improving initial Coulombic efficiency and cycling performance in all‐solid‐state batteries.

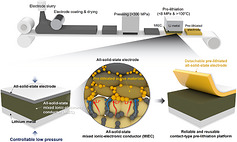

## 1 Introduction

Driven by the rapid and anticipated growth of electric vehicles, robotics, and urban air mobility, the demand for energy storage systems that offer both high energy density and intrinsic safety have become increasingly critical. All‐solid‐state batteries, which replace flammable liquid electrolytes with solid electrolytes, are expected to meet these requirements [1, 2, 3, 4, 5, 6]. Although several solid electrolytes have recently been reported to exhibit limited thermal stability and their safety has therefore been extensively assessed, they still offer the potential to achieve non‐ or low‐flammability depending on their composition [7, 8, 9, 10, 11]. This property significantly reduces the risk of thermal runaway and ignition under abuse conditions. In addition, the non‐flowing nature of solid electrolytes enables precise spatial control, thereby allowing for bipolar stacking of unit cells [12, 13]. The energy density of bipolar‐stacked cells can be improved without altering the active material chemistry employed in conventional lithium‐ion batteries.

To further enhance the energy density of all‐solid‐state batteries, lithium‐metal and anode‐less electrode configurations are widely regarded as promising strategies, as they can substantially reduce the overall weight and volume of the cells [14, 15, 16, 17, 18, 19]. In such systems, it has conventionally been assumed that mechanically robust solid electrolyte layers provide sufficient strength to suppress dendritic lithium growth, thereby enabling the reversible utilization of lithium metal. However, even in the presence of solid electrolyte layers, lithium‐metal chemistry inevitably induces dendritic growth, which critically undermines cell reliability [20, 21, 22, 23, 24]. Furthermore, the intrinsically limited lithium‐ion diffusivity within lithium metal under ambient conditions promotes void formation at the interface between lithium metal and the solid electrolyte layer during repeated cycling [25, 26, 27, 28, 29, 30]. This process induces current constriction at progressively shrinking contact regions, thereby accelerating dendrite nucleation and propagation. Therefore, conventional anodes composed of active material particles should remain under consideration as a more reliable and practical alternative.

Another important consideration in all‐solid‐state batteries lies in the narrow electrochemical stability window of solid electrolytes. Many promising solid electrolytes are unstable within the anode potential range and undergo electrochemical decomposition during the initial cycles, irreversibly consuming lithium ions transported from the cathode [31, 32, 33, 34, 35]. This reaction decreases the quantity of lithium available for cycling, thereby overburdening the cathode particles and ultimately reducing the cell performance. For example, the widely studied sulfide‐based solid electrolyte Li_6_PS_5_Cl (LPSCl) exhibits a particularly narrow electrochemical stability window of 1.25–2.50 V (vs. Li/Li^+^) [35]. Despite its poor intrinsic stability, LPSCl‐based all‐solid‐state batteries can operate stably because their decomposition products, including Li_3_P, P, Li_2_S, and LiCl, are electrochemically stable [32]. These products suppress further decomposition of LPSCl while maintaining efficient lithium‐ion conduction at the interface. Nevertheless, the irreversible capacity loss incurred during the initial decomposition must be minimized and compensated through appropriate strategies [36].

Pre‐lithiation of active materials prior to cycling is an effective strategy to compensate for initial capacity loss [37, 38, 39, 40, 41, 42]. Several approaches have been proposed, including chemical pre‐lithiation with molecularly tailored complexes, lithium‐metal‐assisted pre‐lithiation, and cathode pre‐lithiation. However, most of these methods have been developed for lithium‐ion batteries employing liquid electrolytes, whereas pre‐lithiation strategies specifically tailored for all‐solid‐state batteries remain relatively unexplored. In all‐solid‐state systems, interfacial properties critically influence lithium‐ion transport between solid components because of the relative inefficiency of solid‐solid contact, compared to conformal liquid–solid contact in lithium‐ion batteries. For this reason, unintended interface modification caused by liquid‐based pre‐lithiation methods can adversely affect lithium‐ion transport at the interfaces. To confirm this, a surface‐modified graphite electrode prepared through an electrochemical formation step in a liquid electrolyte was evaluated in an all‐solid‐state system (Figure S1). During the formation step, a solid electrolyte interphase (SEI) develops on the graphite surface, with its characteristics depending on the liquid electrolyte composition [39, 43]. In general, this SEI can become an obstacle by forcing lithium ions to take detoured diffusion pathways between graphite particles [44]. Therefore, as shown in Figure S1, the pristine graphite electrode exhibits superior rate performance as an all‐solid‐state anode, whereas the liquid electrolyte derived SEI‐modified graphite electrode shows significantly increased overpotential and substantial capacity decay at high C‐rates. These results indicate that a pre‐lithiation strategy tailored for all‐solid‐state batteries must be carefully designed with explicit consideration of interfacial properties.

In this work, we present a reliable and reusable all‐solid‐state contact‐type pre‐lithiation platform that utilizes a mixed ionic–electronic conductor (MIEC) comprising solid electrolyte and electroconductive agent particles. In this approach, lithium ions and electrons are simultaneously transported from lithium metal to the target electrode through the MIEC in dry contact, enabling precise lithiation and delicate surface modification of the all‐solid‐state electrode. The degree of pre‐lithiation can be effectively controlled by adjusting contact time, pressure, and temperature. We demonstrate that the resulting uniform pre‐lithiation throughout the entire electrode particles leads to excellent initial Coulombic efficiency (ICE) and stable cycling performance. Notably, successful pre‐lithiation with minimal interfacial impedance was achieved under a low stack pressure of 8 MPa, a practically relevant level significantly lower than the pressures typically used for cell fabrication (> 300 MPa). The pre‐lithiated electrodes can be directly employed in all‐solid‐state battery fabrication without any additional post‐treatment, and the reusability and process reproducibility of the pre‐lithiation platform using the MIEC were also verified.

## 2 Results and Discussion

### 2.1 Mechanism of All‐Solid‐State Pre‐Lithiation using MIEC

**FIGURE 1:**
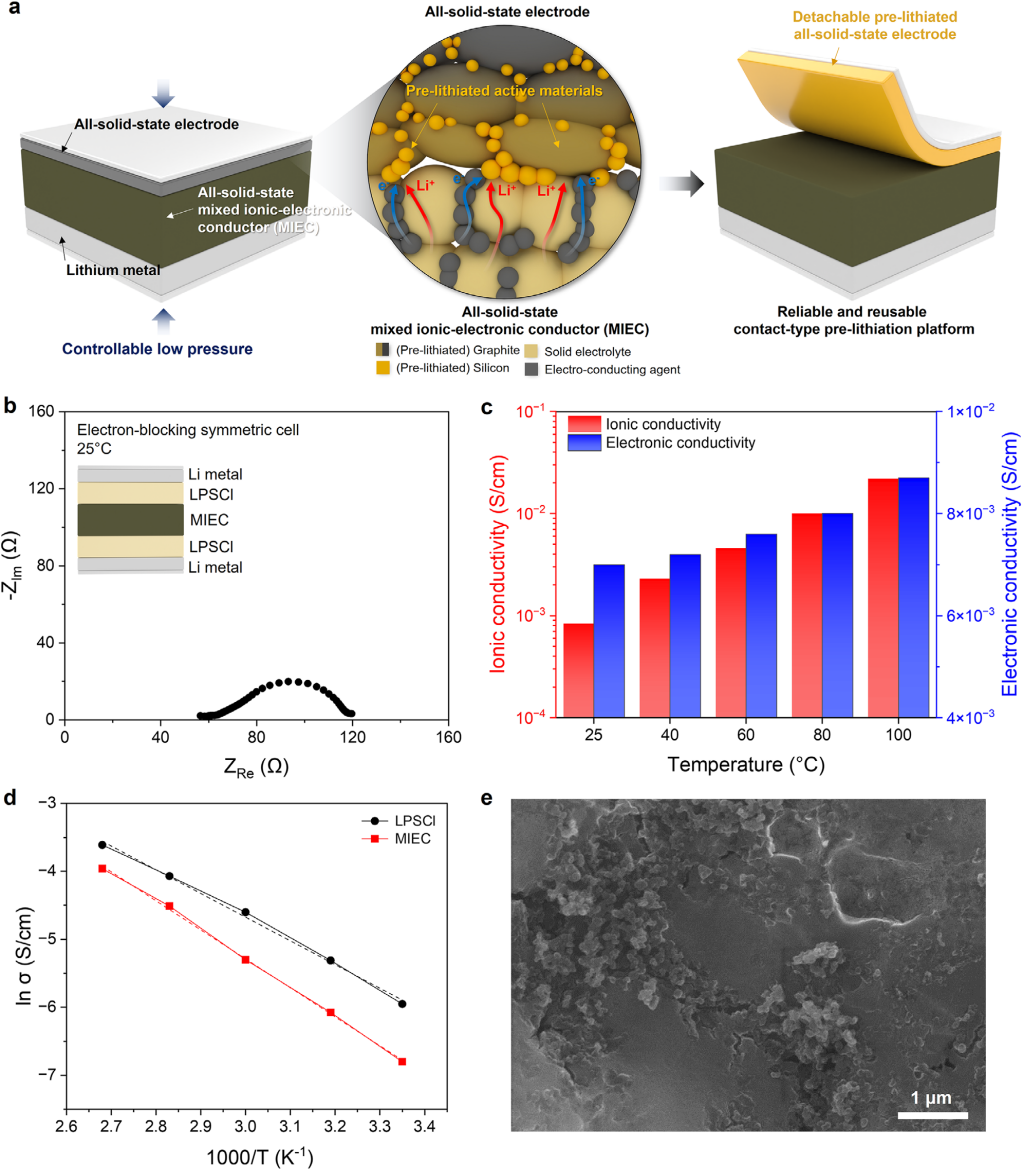
Concept and transport characteristics of MIEC. (a) Schematic illustration of a pre‐lithiation platform based on all‐solid‐state MIEC, along with its working mechanism. (b) Nyquist plots for an electron‐blocking symmetric cell with MIEC (Li | solid electrolyte | MIEC | solid electrolyte | Li). (c) Summary of ionic and electronic conductivities as a function of temperature for MIEC. (d) Arrhenius plots of LPSCl and MIEC. (e) Top‐view SEM image of MIEC surface.

Figure 1a illustrates a pre‐lithiation platform that operates through all‐solid‐state charge carrier transport, along with its underlying mechanism. The system, comprising thick lithium metal and MIEC, leverages the chemical potential difference between the lithium metal and the target electrode, which are interconnected via both ionic and electronic pathways. When the solid‐state electrode, which generally exhibits a lower chemical potential than lithium metal, comes into contact with the surface of the MIEC under controlled pressure, lithium ions and electrons are simultaneously transported from the lithium metal to the electrode until the electrochemical potential difference is balanced. Accordingly, the degree of pre‐lithiation can be precisely controlled by adjusting experimental parameters such as contact time, stack pressure, and operating temperature. Upon completion of pre‐lithiation under these controlled conditions, the pre‐lithiated electrode can be easily detached from the MIEC surface and directly employed in the fabrication of all‐solid‐state batteries without the need for any additional post‐treatment. Furthermore, the MIEC is reusable and mechanically stable, offering significant advantages in terms of process efficiency. When implementing conventional pre‐lithiation using liquid electrolytes, careful consideration must be given to the effects of solvent evaporation on chemical reactivity, as well as to the system design required to ensure consistent pre‐lithiation performance [45]. In contrast, all‐solid‐state systems can operate stably over a wide temperature range and maintain consistent chemical activity without changes in composition.

As a proof‐of‐concept electrode for pre‐lithiation, a diffusion‐dependent electrode (DDE) was employed. The DDE is composed almost entirely of active material and relies on interparticle diffusion between active material particles for charge and discharge [46, 47, 48]. Graphite and silicon have been validated as suitable active materials for this electrode configuration. During the pre‐lithiation process, the DDE enables lithium‐ion diffusion throughout the entire electrode until the internal lithium concentration gradient is fully equilibrated [42]. This results in a highly uniform degree of pre‐lithiation across the electrode, effectively preventing over‐lithiation of specific active material particles. In this study, a graphite/nanometer‐scale silicon (Gr/n‐Si; Gr:n‐Si = 90:10, wt.%) DDE was primarily used [46]. Although the pre‐lithiation strategy was initially demonstrated using the DDE, it is not limited to this electrode architecture and can be readily extended to conventional composite electrodes, as discussed in a later section.

For the formation of the MIEC, LPSCl and carbon black (CB) were homogeneously mixed and pelletized, serving as the ionic and electronic conductors, respectively. To achieve balanced transport capability for lithium ions and electrons in the MIEC, the blending ratio of LPSCl to CB was carefully optimized. The transport properties of the composites were evaluated by impedance analysis using an ion‐blocking symmetric cell configuration composed of current collector | MIEC | current collector (Figure S2). When the LPSCl/CB ratio was 97/3 wt.%, the CB content was insufficient to form a percolating electronic network, and accordingly, conventional capacitive behavior was observed. In contrast, increasing the CB content to 7 wt.% led to the composite exhibiting electronically resistive characteristics due to the predominance of electronic conduction. At a 95/5 wt.% composition, the composite exhibited a semicircular impedance response, indicating balanced mixed conduction. Based on these observations, the LPSCl/CB ratio was optimized to 95/5 wt.% in the present study, which can be adjusted depending on the type of ionic/electronic conductor used. To systematically investigate the ionic and electronic conductivities of the MIEC, temperature‐dependent impedance spectroscopy was performed using an electron‐blocking symmetric cell configuration (Figure 1b and Figure S3). The ionic and electronic conductivities of the MIEC as a function of temperature were derived from the equivalent circuit model with frequency analysis (Figure 1c). While the electronic conductivity slightly increases from 7.0 × 10^−3^ to 8.7 × 10^−3^ S cm^−1^ (a 24.3% improvement), the ionic conductivity increases rapidly with rising temperature due to thermal activation of lithium‐ion transport. Compared to the pellet composed solely of LPSCl, the MIEC exhibits reduced ionic conductivity and slightly increased activation energy, which is attributed to the structural distortion caused by CB (Figure 1d and Figure S4). At temperatures above 60°C, the MIEC exhibits ionic conductivity comparable to its electronic conductivity, as shown in Figure 1c. Figure 1e shows a scanning electron microscopy (SEM) image of the MIEC surface that reveals a homogeneous distribution of LPSCl and CB, which contributes to the uniform pre‐lithiation of the electrode.

### 2.2 Process Optimization for All‐Solid‐State Pre‐Lithiation

**FIGURE 2:**
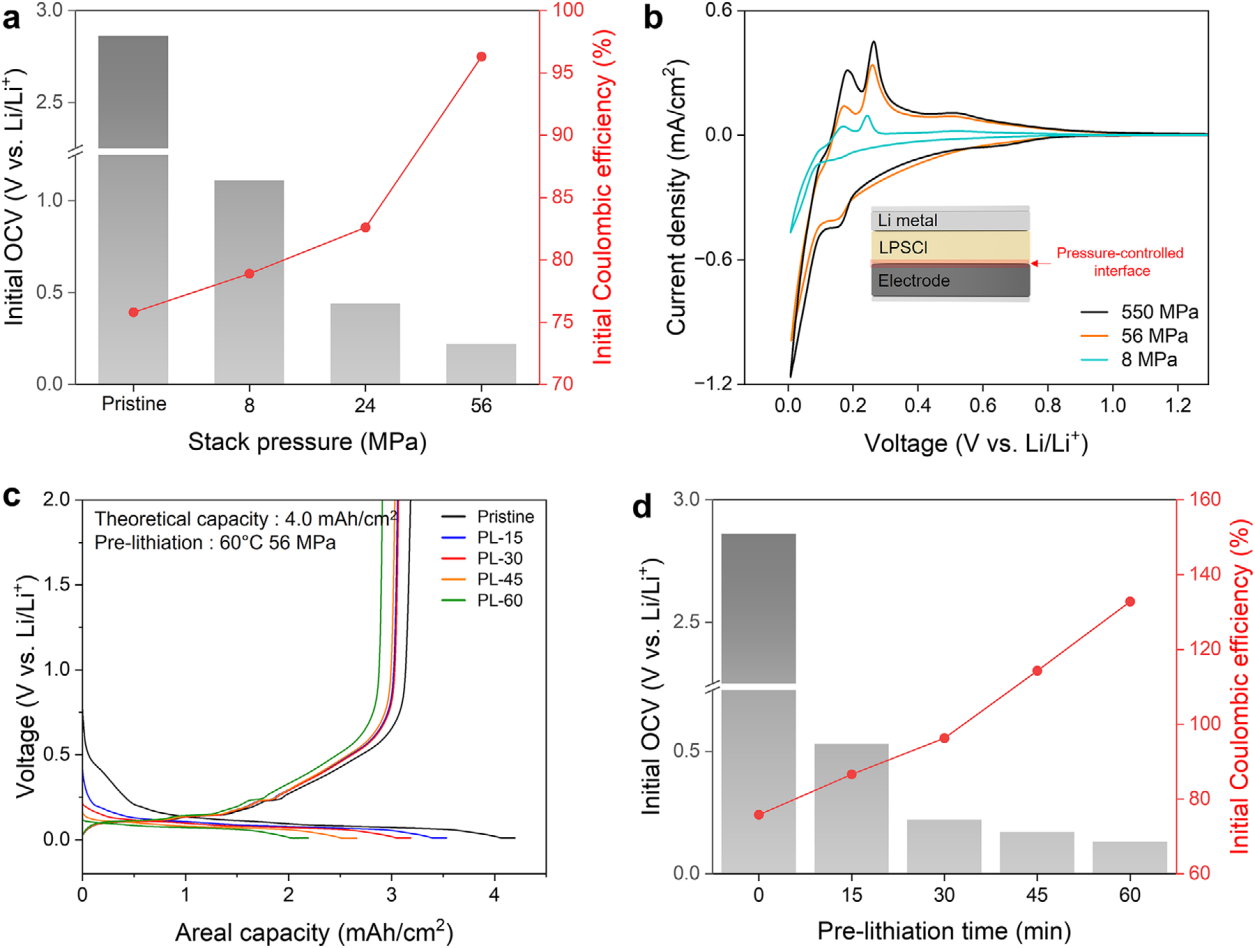
Electrochemical characteristics of pressure‐ and time‐controlled pre‐lithiation. (a) ICE and OCV of Gr/n‐Si DDE half‐cells pre‐lithiated at 60°C for 30 min as a function of stack pressure. (b) Cyclic voltammetry measurements of Gr/n‐Si DDE | Li half‐cells under various stack pressures. (c) First cycle voltage profiles of the pristine DDE and PL‐series half‐cells pre‐lithiated under 56 MPa at 60°C. (d) ICE and OCV of Gr/n‐Si DDE half‐cells pre‐lithiated under 56 MPa at 60°C as a function of pre‐lithiation time.

To assess the feasibility of the pre‐lithiation method using the all‐solid‐state MIEC, systematic experiments were performed under varying pressure and time conditions. As an initial step, the detachability of the target electrode from the MIEC was evaluated at different applied stack pressures, as this is a crucial factor in the reusability of the proposed pre‐lithiation process. As shown in Figure S5, the Gr/n‐Si DDE was easily detached from the MIEC within the tested pressure range, and no notable structural deformation was macroscopically observed in both the electrode and MIEC. To investigate the effect of stack pressure on pre‐lithiation behavior, Gr/n‐Si DDEs were pre‐lithiated at 60°C for 30 min under stack pressures of 8, 24, and 56 MPa, and the cell properties were measured in Gr/n‐Si DDE | Li half‐cell at 60°C. Figure 2a presents the ICE and open‐circuit voltage (OCV) under each pressure condition. The corresponding voltage profiles are shown in Figure S6. Pre‐lithiation was successfully achieved under all tested conditions, as evidenced by a decrease in the first discharge capacity and initial OCV, along with an increase in ICE. Cyclic voltammetry (CV) results for the pristine and pre‐lithiated DDEs distinctly indicate the absence of initial decomposition of LPSCl in the pre‐lithiated DDE, suggesting that pre‐lithiation not only supplies lithium to the active materials but also contributes to the formation of a SEI (Figure S7). Additionally, the electrochemical behavior varied significantly with the applied stack pressure. As the stack pressure increased, the OCV continued to decrease, while the ICE showed an increasing trend. This behavior is attributed to enhanced interfacial contact between the MIEC and DDE under higher pressure, which facilitates more efficient lithium transfer. Notably, the DDE pre‐lithiated at 56 MPa for 30 min achieved the highest ICE of 96.3%. It is also worth noting that successful pre‐lithiation was achieved even under a relatively low pressure of 8 MPa, which is significantly lower than the typical cell fabrication pressure (> 300 MPa).

To elucidate the origin of pre‐lithiation under such low stack pressures, the overall ionic and electronic transport properties at the interface were evaluated as a function of stack pressure. Figure S8 and Table S1 present the impedance results obtained from an ion‐blocking symmetric cell configuration, in which electronic conduction can be assessed by measuring impedance in the low‐frequency region. When a conventional fabrication pressure of 550 MPa was applied, the interfacial resistance exhibited a very low resistance of 0.16 Ω cm^2^, which is attributed to the formation of a conformal interface. However, under this condition, the all‐solid‐state electrode is not easily or cleanly separable from the MIEC. Under much lower stack pressure, relatively low interfacial resistance was still observed as 18.5 Ω cm^2^ at 8 MPa. These results suggest that electronic conductivity remains sufficient for pre‐lithiation even under such low‐pressure conditions. On the other hand, evaluating ionic transport at the interface as a function of stack pressure was challenging in an electron‐blocking symmetric cell configuration, as it is difficult to reliably form such a cell with different interface stack pressures. Therefore, CV measurements of the DDE half‐cells were conducted under various stack pressures to indirectly probe ionic transport behavior (Figure 2b and Figure S9). Figure 2b presents the first‐cycle CV profiles of the DDEs under various stack pressures. Consistent with the electronic conductivity results, the DDE with a conformal interface exhibited facile lithium‐ion transfer. In the cell under 8 MPa, the redox peaks were reduced in intensity but remained well defined and reversible, indicating efficient interfacial ion transport. These results suggest that both electronic and ionic transport are sufficient to enable pre‐lithiation, even when the interface between the electrode and the MIEC is loosely formed.

To further evaluate the temporal progression of pre‐lithiation, the process was conducted at 60°C under a pressure of 56 MPa for varying durations: 15, 30, 45, and 60 min (denoted as PL‐15, PL‐30, PL‐45, and PL‐60, respectively). Figure 2c presents the first cycle voltage profiles of the pristine DDE and the PL‐series half‐cells. The first discharge capacities of the pristine, PL‐15, PL‐30, PL‐45, and PL‐60 DDEs were 4.19, 3.53, 3.18, 2.66, and 2.19 mAh cm^−2^, respectively, indicating a clear decrease as pre‐lithiation time increased. The corresponding voltage profiles for 3 cycles are presented in Figure S10. Figure 2d displays the OCV and ICE for all samples, demonstrating an inverse correlation between OCV and ICE. When the pre‐lithiation duration exceeded 30 min, the ICE surpassed 100%, representing a substantial improvement compared to the bare DDE (75.8%). These results confirm that the degree of pre‐lithiation can be precisely modulated by adjusting the contact time, exhibiting a linear relationship between pre‐lithiated capacity and contact duration (Figure S11).

Furthermore, the spatial uniformity of pre‐lithiation was validated through surface observations. Optical microscopy (OM) analysis reveals that the electrode surface color changed uniformly from dark brown to reddish and eventually to yellow as the pre‐lithiation time increased (Figure S12) [49]. This color transition was more distinctly observed in the DDE composed solely of graphite, as shown in Figure S13. Also, the cross‐sectional OM image shows a homogeneous color transition across the entire electrode thickness, and the color remains stable over time, supporting the depth‐wise uniformity and stability of lithiation (Figure S14). Because the pre‐lithiation process is driven by the chemical potential difference with lithium metal, both silicon and graphite are, in principle, amenable to lithiation. To validate this, a DDE composed of n‐Si was also fabricated and subjected to the same pre‐lithiation procedure. Figure S15 displays OM images and voltage profiles of the pristine and pre‐lithiated n‐Si DDEs. The pristine electrode surface exhibited a brownish color, which turned uniformly gray after pre‐lithiation. Electrochemical evaluation of half‐cells using pristine and pre‐lithiated n‐Si DDEs revealed an increase in ICE from 71.2% to 117.7%, confirming that lithiation also occurred through the silicon particles.

In addition to the in‐plane uniformity verified by OM analysis, the out‐of‐plane uniformity of pre‐lithiation was confirmed by monitoring the OCV and observing surface changes of the PL‐60 graphite DDE over time at 25°C (Figure S16). Initially, the OCV of the as‐prepared PL‐60 was 0.105 V, and the electrode surface exhibited a uniform reddish‐yellow color. As the rest time increased, the OCV gradually rose to approximately 0.125 V, accompanied by a progressive darkening of the electrode surface. This evolution indicates that lithium ions initially concentrated near the surface in contact with MIEC diffused into the bulk of the electrode through interparticle pathways. This process mitigates the risk of surface over‐lithiation of active material particles and contributes to electrochemically stable operation.

**FIGURE 3:**
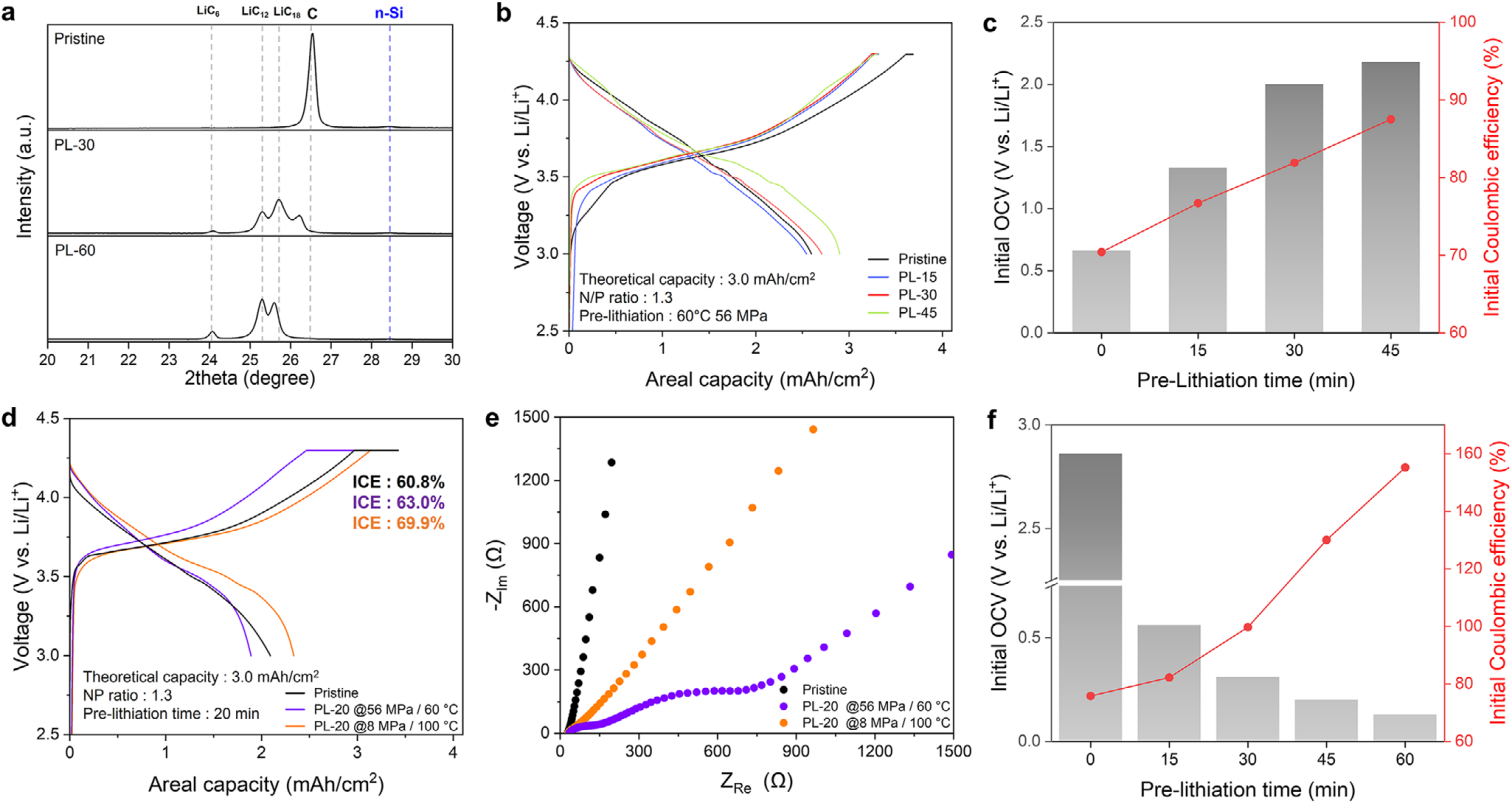
Interfacial and electrochemical evolution during contact‐dependent pre‐lithiation. (a) X‐ray diffraction result as a function of pre‐lithiation time for Gr/n‐Si DDEs. (b) First‐cycle voltage profiles of the pristine DDE and PL‐series full‐cell at 60°C. (c) ICE and OCV of Gr/n‐Si DDE full cells as a function of pre‐lithiation time (d) First‐cycle voltage profiles of the pristine DDE and PL‐series full cell at 25°C. (e) Nyquist plot results of pristine DDE and PL‐series symmetric cells. (f) ICE and OCV of Gr/n‐Si DDE full cells pre‐lithiated under 8 MPa at 100°C as a function of pre‐lithiation time.

X‐ray diffraction (XRD) was employed to analyze the bulk structural evolution of graphite and silicon. Figure 3a and S17 show the XRD results of the pristine DDE and pre‐lithiated DDEs. In the pristine DDE, characteristic peaks were observed at 2θ = 26.8° and 28.6°, corresponding to graphite and silicon, respectively [50]. Upon pre‐lithiation, the intensity of the graphite peak at 26.8° gradually decreased, while new diffraction peaks emerged at 2θ = 25.8°, 25.4° and 24.1°, corresponding to LiC_18_, LiC_12_ and LiC_6_, respectively [51]. After 60 min of pre‐lithiation, the graphite peak disappeared entirely, indicating that most of the graphite particles were lithiated. In contrast, no distinct XRD peaks were observed for pre‐lithiated n‐Si, likely due to nanoscale particle size and low crystallinity. Moreover, the crystalline structure of silicon is known to become amorphous upon lithiation, making it difficult to detect using XRD (Figure S17a) [52]. Nevertheless, cross‐sectional SEM images of the lithiated electrode revealed pronounced volume expansion of silicon, as evidenced by the overall increase in electrode thickness and visible swelling of n‐Si regions situated between graphite particles (Figure S18).

To confirm the enhancement effect of pre‐lithiation, the electrochemical performance of all‐solid‐state full cells incorporating pristine DDE, PL‐15, PL‐30, and PL‐45 electrodes was evaluated. For the full cell configuration, a cathode composed of LiNi_0.6_Co_0.2_Mn_0.2_O_2_ (NCM622) and LPSCl in a weight ratio of 7:3 was prepared using a conventional slurry‐casting method. Figure 3b,c presents the voltage profiles, OCVs, and ICEs during the first cycle. Compared to the full cell with the pristine DDE, the pre‐lithiated cells exhibited a consistent decrease in charge capacity and a stepwise increase in discharge capacity with increasing pre‐lithiation time. Therefore, the ICE of the pristine DDE‐based full cell was 70.4%, whereas those of the pre‐lithiated DDEs significantly improved to 76.7%, 81.9%, and 87.5% for PL‐15, PL‐30, and PL‐45, respectively. For PL‐45, a lithium stripping plateau was observed in the discharge profile, indicating excessive pre‐lithiation, which is consistent with the high ICE of 114% observed in the half‐cell test (Figure 2d). In addition, although the ICE of the half‐cell exceeded 100%, the ICE of the corresponding full cell was limited to 87.5%. This discrepancy is attributed to the cathode‐limited nature of the system, in which the maximum achievable ICE through pre‐lithiation is constrained by the ICE of the cathode [53]. To verify this, the cathode was replaced with a LiCoO_2_/Li_3_InCl_6_ composite cathode exhibiting a higher ICE of 95.2%, instead of NCM622 with ICE of 88.2% (Figure S19). In the resulting full cell, the ICE reached 94.3%, which is higher than that of the full cell with NCM622, despite identical pre‐lithiation conditions and their comparable areal capacities. This result confirms that the ICE of the pre‐lithiated full cell is fundamentally influenced by the cathode.

To broaden the practical applicability of this pre‐lithiation method, it is essential to ensure stable full‐cell operation with a pre‐lithiated electrode at ambient temperatures, where lithium‐ion kinetics s considerably slower than at elevated temperatures. However, as shown in Figure 3d, the full cell incorporating the pre‐lithiated electrode exhibited relatively high internal resistance. Compared with the full cell containing the pristine Gr/n‐Si DDE, the PL‐20 pre‐lithiated under 56 MPa at 60°C cell showed a higher overpotential and reached the cut‐off voltage earlier, accompanied by an extended constant‐voltage charging period. Impedance analysis of symmetrical cell configuration with pristine and pre‐lithiated electrodes provided consistent results (Figure 3e). Unlike the pristine DDE, which exhibited simple capacitive behavior, the PL‐20 displayed a pronounced semicircle indicative of high interfacial resistance, along with Warburg behavior characterized by a straight line with a 45° slope in the low‐frequency region. To elucidate the origin of the high resistance, x‐ray photoelectron spectroscopy (XPS) was conducted on the pre‐lithiated DDEs. Figure S20 presents the XPS spectra of the DDEs as a function of pre‐lithiation time. Consistent with previous results, a peak corresponding to Li_x_C appeared near 283 eV in the C 1s spectrum after pre‐lithiation [54, 55]. In addition, a peak around 289–290 eV became more pronounced, and its intensity increased with longer pre‐lithiation durations [56, 57]. This peak is most likely attributed to the formation of Li_2_CO_3_, as also suggested by the Li 1s spectrum [58]. This oxidation component may be caused by reaction with lithium within lithiated Gr/Si and oxygen‐containing functional groups of CB [59, 60]. These decomposition products generally exhibit low ionic conductivity, which may account for the increased resistive behavior observed in Figure 3d,e [61, 62].

In this regard, the formation of such decomposition products must be minimized to ensure facile lithium‐ion transport at the interface. To this end, the pre‐lithiation of the DDE was performed under a relatively low stack pressure of 8 MPa, as decomposition products are likely to form locally at the small contact interface. However, under this low stack pressure condition, although pre‐lithiation occurs, its efficiency remains limited due to the reduced electrochemically active area (Figure 2a). As shown in Figure S21, when pre‐lithiation was carried out under 8 MPa at 60°C, the ICE did not show significant improvement over time. To overcome this limitation, the pre‐lithiation temperature was elevated to 100°C to promote faster lithium‐ion kinetics. In contrast to conventional MIECs with a liquid‐phase conductor, the all‐solid‐state MIEC enables pre‐lithiation over a wide temperature range and exhibits enhanced ionic/electronic transport at elevated temperatures (Figure 1c). This approach effectively enhances the pre‐lithiation efficiency, as demonstrated in Figure 3f. Furthermore, to investigate the lithium‐ion kinetics at 100°C, CV measurements were conducted under a stack pressure of 8 MPa. As shown in the CV profile in Figure S22, despite the low stack pressure, a reduction current of −5.17 mA cm^−^^2^ was observed, indicating a remarkably rapid reaction. This value is substantially larger than that obtained at room temperature under the same stack pressure (−0.47 mA cm^−2^) (Figure 2b). As a result, the impedance response of the DDE pre‐lithiated under 8 MPa at 100°C showed significant improvement compared to that at 56 MPa and 60°C for the same duration. The distinct semicircle observed for the DDE pre‐lithiated under 56 MPa at 60°C was completely suppressed (Figure 3e). Correspondingly, the constant‐voltage charging period was shortened, and the ICE of the full cell improved from 63.0% to 69.9% (Figure 3d). These results clearly indicate that controlling the contact area during pre‐lithiation can significantly influence interfacial properties (Figure S23), which are closely associated with overall cell performance. To further elucidate these effects, depth‐profiling XPS analysis was performed on electrodes pre‐lithiated under two distinct conditions: 56 MPa at 60°C and 8 MPa at 100°C (Figure S24). As illustrated in Figure S24, the Li_2_CO_3_ content is significantly lower for the electrode pre‐lithiated under 8 MPa at 100°C compared to under 56 MPa at 60°C. Following 10 min of etching, the Li_2_CO_3_ peak is substantially attenuated under 8 MPa at 100°C, whereas it remains clearly discernible under 56 MPa at 60°C. Furthermore, under high‐pressure conditions, the Si 2p signal is largely obscured by decomposition products, with persistent noise observed even after 30 min of etching. In contrast, under 8 MPa at 100°C, the Si peak becomes progressively more distinct with etching. These findings are consistent with the Li 1s spectra, collectively indicating that the 8 MPa/100°C condition minimizes the effective interfacial contact area between the MIEC and the electrode, thereby effectively suppressing the formation of parasitic byproducts.

In the half‐cell test, the ICE of the DDE pre‐lithiated under 8 MPa at 100°C for 30 min reached 99.7%, which is comparable to that obtained under 56 MPa at 60°C for 30 min (96.3%) (Figure 2d) and markedly higher than the value achieved under 8 MPa at 60°C for 30 min (74.0%) (Figure S21). The corresponding voltage profiles are shown in Figure S25. Under these conditions, a linear relationship between pre‐lithiated capacity and contact duration was also confirmed (Figure S11). The slope of pre‐lithiated capacity versus contact duration was higher at 8 MPa and 100°C (0.041) than at 56 MPa and 60°C (0.034).

### 2.3 Digital Twin Simulation of Pre‐Lithiation Behavior

Digital twin simulations were conducted to quantitatively elucidate the interfacial contact characteristics and pre‐lithiation behavior between the graphite DDE and the MIEC under varying stack pressures. Virtual microstructures of the DDE and MIEC were generated based on the particle size distributions, morphologies, and volume fractions of graphite, PVDF, LPSCl, and CB (Figure S26) [63]. The DDE structure was then stacked onto the MIEC structure, and compression simulations were performed under stack pressures of 8, 24, 40, 56, and 72 MPa, accounting for the mechanical properties of each constituent material (Tables S2 and S3). Further details regarding the structure generation procedure and simulation methodology are provided in the supplementary information.

**FIGURE 4:**
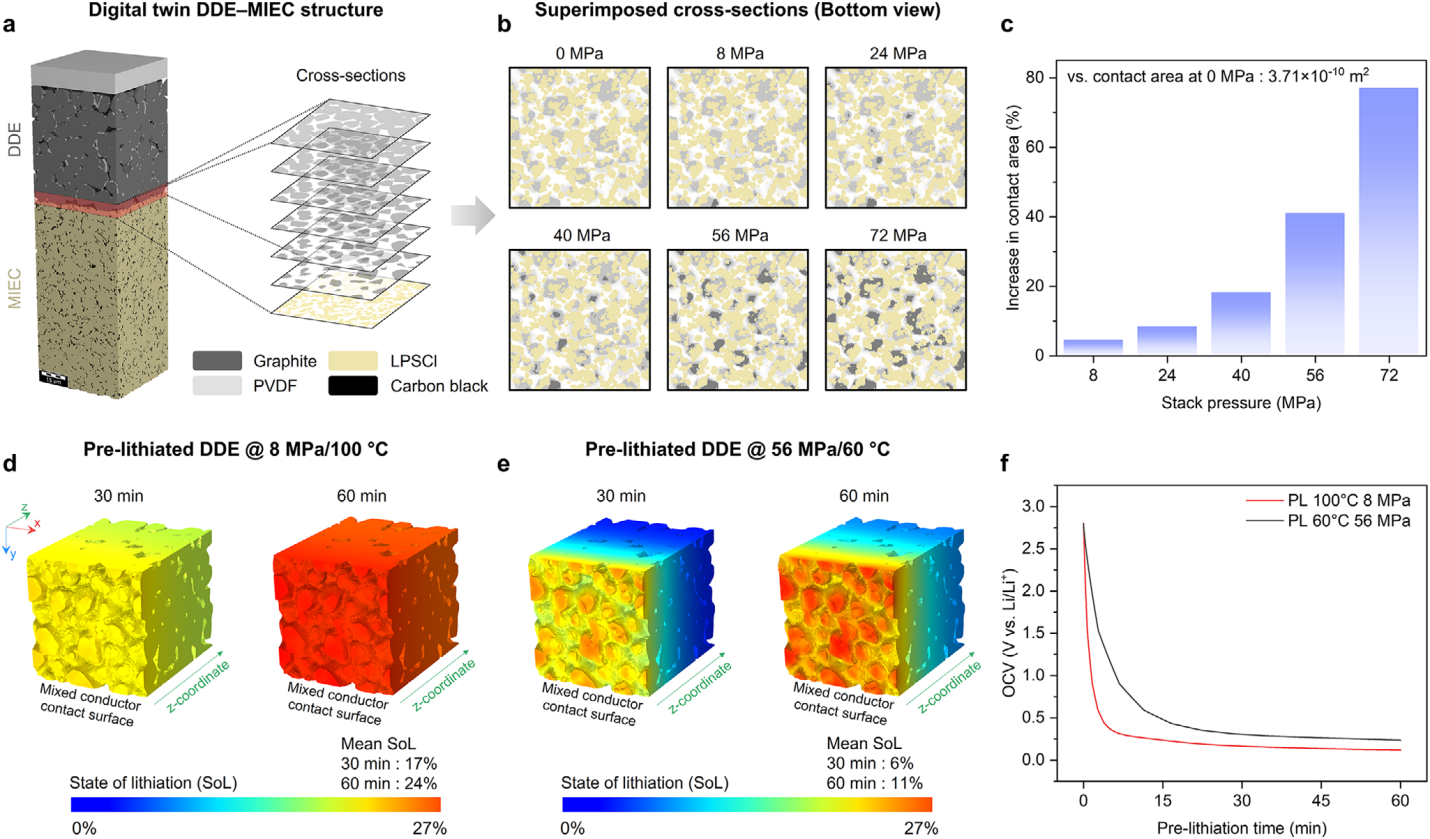
Digital twin simulation of pre‐lithiation behavior. (a) Digital twin DDE–MIEC structure and a series of two‐dimensional cross‐sectional images extracted at 0.4 µm intervals along the interface. (b) Bottom‐view superimposed cross‐sectional images obtained under different stack pressures. (c) Increase in interfacial contact area with increasing pressure relative to 0 MPa. SoL distributions after 30 and 60 min under the (d) 8 MPa/100°C and (e) 56 MPa/60°C. (f) Evolution of OCV during pre‐lithiation under 8 MPa/100°C and 56 MPa/60°C.

Multiple cross‐sectional images across the DDE–MIEC interfacial region were extracted and superimposed to generate bottom‐view two‐dimensional images for the quantitative analysis of interfacial contact evolution with increasing stack pressure (Figure 4a,b). The superimposed images clearly illustrate the progressive mechanical interlocking between graphite and LPSCl particles as the stack pressure increases. This interlocking behavior effectively fills interparticle voids at the interface, thereby enhancing the interfacial contact area. Quantitative analysis revealed that the contact area increased by 4.5% (8 MPa), 8.4% (24 MPa), 18.2% (40 MPa), 41.1% (56 MPa), and 71.1% (72 MPa) relative to 0 MPa, exhibiting an exponential growth trend with increasing stack pressure (Figure 4c). Consequently, this increase facilitates efficient pre‐lithiation, which is consistent with the results shown in Figure 2a. Additionally, it is observed that even under a relatively low stack pressure of 8 MPa, the interface, which serves as the primary pathway for pre‐lithiation, is already well developed. This behavior is mainly attributed to locally concentrated stresses generated at particle–particle contact points, even under low applied stack pressures. Figure S27 shows von Mises stresses exceeding the critical yield stress of LPSCl (20–60 MPa) under stack pressures of 8 and 56 MPa [64, 65, 66, 67]. Notably, even at an applied pressure of 8 MPa, which is significantly lower than the macroscopic yield stress, localized contact regions induce substantially elevated partial pressures and mechanical distortion, thereby providing effective pathways for pre‐lithiation.

To further investigate whether comparable pre‐lithiation behavior could be achieved with a limited contact area, reaction kinetics at elevated temperature were evaluated. For systematic comparison, simulations were carried out under 8 MPa at 100°C and under 56 MPa at 60°C. The electrochemical simulation results of Figure 4d,e and Movies S1, S2 visualized the state‐of‐lithiation (SoL) distributions within the three‐ dimensional DDE structures after 30 and 60 min of pre‐lithiation. In addition, Figure S28 presents the SoL values extracted from the simulated structure averaged along the electrode thickness (z‐direction). Under the condition of 8 MPa and 100°C, the average SoL values were 17% (30 min) and 24% (60 min), which were higher than those obtained under the condition of 56 MPa and 60°C (6% and 11%, respectively). Moreover, a pronounced lithium concentration gradient developed along the electrode thickness for the pre‐lithiated DDE under the condition of 56 MPa and 60°C. In contrast, lithiation proceeded more uniformly under the condition of 8 MPa and 100°C, owing to an approximately fourfold increase in the diffusion coefficient at elevated temperature (supplementary information). The OCV evolution during pre‐lithiation was calculated based on the lithium concentration within the DDE over time (Figure 4f). The pre‐lithiated DDE under the condition of 8 MPa and 100°C exhibited a lower OCV than under the condition of 56 MPa and 60°C, indicating a higher SoL throughout all time intervals. Overall, these results indicate that the relatively limited interfacial contact area under 8 MPa can be mitigated by enhanced kinetics at elevated temperatures. As a result, uniform pre‐lithiation can be achieved while minimizing undesired side reactions, which is in strong agreement with the experimental findings.

### 2.4 Electrochemical Performance of Pre‐Lithiated Electrodes

**FIGURE 5:**
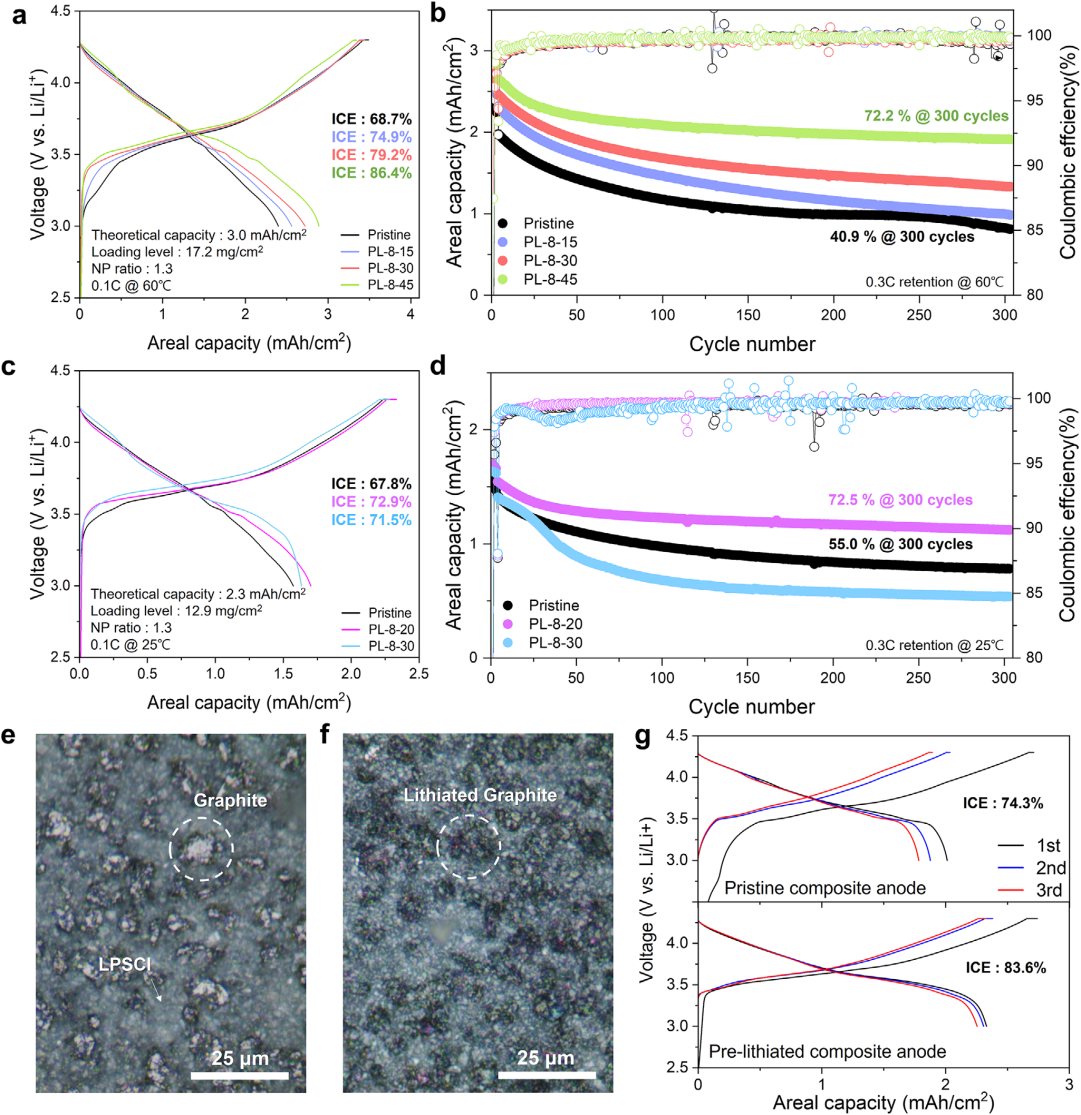
Full‐cell performance and applicability of pre‐lithiation. First cycle voltage profiles of the pristine DDE and PL‐series full‐cell (a) at 60°C and (c) 25°C, pre‐lithiated at 100°C under 8 MPa. Capacity retention and Coulombic efficiency of the pristine DDE and PL‐series full‐cell (b) at 60°C and (d) 25°C with different pre‐lithiation times. Optical image of the (e) pristine and (f) pre‐lithiated graphite composite electrode. (g) Charge‐discharge voltage profile of full cells with pristine and pre‐lithiated graphite composite electrode.

Figure 5 presents the electrochemical performance of full cells tested at 60°C and 25°C, respectively, using pre‐lithiated DDEs prepared under 8 MPa at 100°C. Consistent with the previous pre‐lithiation results, both the ICEs and OCVs of the full cells were effectively controlled by the contact duration (Figure S29). The samples pre‐lithiated for 15, 20, 30, and 45 min under 8 MPa are referred to as PL‐8‐15, PL‐8‐20, PL‐8‐30, and PL‐8‐45, respectively. For full cells operated at 60°C, the ICE values of PL‐8‐15, PL‐8‐30, and PL‐8‐45 were 74.9%, 79.2%, and 86.4%, respectively, all of which represent clear improvements over the 68.7% observed for the full cell with the pristine DDE, as shown in Figure 5a. Due to the relatively low ICE of the NCM half‐cell (75.6%) (Figure S30), the maximum ICE at 25°C was limited to 72.9% for PL‐8‐20 [53]. Nevertheless, this still represents a notable improvement over the pristine cell (67.8%), as shown in Figure 5c. In addition, capacity retention of the full cells over 300 cycles was evaluated at a charge/discharge rate of 0.3C (Figure 5b and Tables S4,S5). All pre‐lithiated cells exhibited higher areal capacities than the pristine cell, and in particular, PL‐8‐45 achieved nearly twice the areal capacity at 300 cycles. Moreover, the pre‐lithiated electrode exhibited superior rate performance compared to the pristine sample (Figure S31). PL‐8‐45, which exhibited a half‐cell ICE of 130%, showed slightly over‐lithiated behavior, as clearly observed in the discharge profile (Figure 5a). Interestingly, this excess lithium can act as an additional lithium reservoir. This is particularly beneficial for the Gr/n‐Si DDE with 10 wt.% silicon, which suffers from low ICEs due to the formation of irreversible lithium–silicon compounds [68]. The additional lithium can compensate for this loss, thereby contributing to improved capacity retention. However, the over‐lithiation behavior must be carefully controlled. Excessive pre‐lithiation did not lead to further improvements in cell performance (Figure S32), likely due to unfavorable interfacial chemistry, which has also been reported in anode‐less all‐solid‐state batteries [14, 20, 26, 30]. Furthermore, under ambient conditions, the benefit of over‐lithiation was limited, presumably due to the combination of poor interfacial chemistry, sluggish lithium kinetics, and suboptimal mechanical properties of lithium metal (Figure S30) [69]. PL‐8‐30 exhibited a rapid capacity fade due to excess lithium, as observed in the room‐temperature cycling performance. In contrast, PL‐8‐20 demonstrated improved cycle stability, confirming its superior long‐term performance (Figure 5d).

To demonstrate the universality of the pre‐lithiation process, a graphite/LPSCl‐based composite anode was used for the pre‐lithiated electrode. The composite anode was prepared by slurry‐casting a mixture of graphite and LPSCl in a 7:3 weight ratio, followed by pre‐lithiation at 100°C and 8 MPa for 30 min. Despite the presence of additional LPSCl, interparticle diffusion among the graphite particles remained effective, and facile electrode separation was observed. As shown in Figure 5e,f, the OM images reveal clear surface changes in the composite anode after pre‐lithiation. The graphite particles, which initially appeared gray, turned purplish after pre‐lithiation, visually confirming the occurrence of the pre‐lithiation process. Figure 5g presents the voltage profiles over the first three cycles with and without pre‐lithiation. The cell with the pre‐lithiated composite anode exhibited an improved ICE, increasing from 74.2% to 85.2%. These results indicate that the proposed pre‐lithiation platform is not limited to DDEs but can also be broadly applied to various electrode systems. Furthermore, it is worth noting that previous liquid‐based pre‐lithiation processes could influence the surface or bulk properties of chemically vulnerable LPSCl, leading to degradation in cell performance. However, our all‐solid‐state strategy completely eliminates these concerns, preserving LPSCl in its intact form during pre‐lithiation.

To further evaluate the reusability and reliability of the all‐solid‐state pre‐lithiation method, the initial electrochemical performance of five PL‐30 and five PL‐45 electrodes was compared (Figure S33 and Table S6). In both groups, the initial OCV, charge–discharge capacities, and ICEs remained within a consistent range, demonstrating that the MIEC‐based pre‐lithiation process offers high reproducibility with minimal variation. Moreover, the mechanical and chemical stability of the MIEC was evaluated after repeated pre‐lithiation (Figure S34). After five repeated uses, XRD analysis showed no significant change compared to the pristine sample, indicating that the crystal structure of MIEC is well preserved. EIS results showed a slight increase in resistance, but the change was minimal, suggesting that MIEC preserves its intrinsic mixed ionic and electronic conductivity. SEM analysis also confirmed that the surface morphology remains stable and intact. The MIEC retains consistent electrochemical and structural properties even after multiple cycles, underscoring the exceptional reusability and robustness of the MIEC platform. In addition, the scalability of the MIEC was evaluated by fabricating a large‐area MIEC via a slurry‐coating process (Figure S35). Impedance analysis of the slurry‐coated MIEC revealed an impedance profile comparable to that of the conventional pellet‐type MIEC, indicating that the ionic and electronic pathways were well preserved. SEM and EDS images further confirmed that LPSCl and CB were uniformly dispersed throughout the composite. Moreover, the comparative analysis presented in Table S7 demonstrates that the MIEC‐based pre‐lithiation strategy in this work offers practical advantages over existing methods in terms of controllability, reusability, and process scalability.

## 3 Conclusion

In summary, we have demonstrated a reliable and reusable contact‐type pre‐lithiation platform that employs an all‐solid‐state MIEC. The MIEC, composed of LPSCl and CB, provides a well‐balanced combination of ionic and electronic conductivities at an optimized composition. This property enables efficient lithium migration from the lithium metal to the target electrode, driven by their chemical potential difference. The degree of pre‐lithiation can be precisely controlled by adjusting the contact time, stack pressure, and temperature. Notably, efficient pre‐lithiation within a practical processing timeframe can be achieved at low stack pressures and elevated temperatures, with minimal interfacial modification. The pre‐lithiated electrode can be cleanly separated from the MIEC without structural damage, enabling its immediate use without additional treatment and allowing reliable reuse of the MIEC‐based pre‐lithiation platform. Owing to the precise controllability of this method, the pre‐lithiated electrodes exhibited improved ICE and enhanced overall cell performance at both elevated and ambient temperatures. Furthermore, the applicability of this platform was validated for various anodes, including DDEs and composite electrodes. Its straightforward configuration is compatible with scalable film‐forming processes, which demonstrates the practical feasibility of this approach. Based on these results, we believe that the proposed MIEC‐based pre‐lithiation platform will contribute to the realization of high‐performance all‐solid‐state batteries and stimulate further research toward the development of versatile and practical pre‐lithiation strategies.

## Conflicts of Interest

The authors declare no conflicts of interest.

## Supporting information




**Supporting File 1**: adma74035‐sup‐0001‐SuppMat.docx.

**Supporting File 2**: adma74035‐sup‐0002‐MovieS1.mp4.

**Supporting File 3**: adma74035‐sup‐0003‐MovieS2.mp4.

## Data Availability

The data that support the findings of this study are available from the corresponding author upon reasonable request.
