# Supplementary material for: Reliable and Reusable All‐Solid‐State Contact‐Type Pre‐Lithiation Platform for High‐Performance All‐Solid‐State Batteries
Source: Adv Mater. 2026 Jul 8;38(46):e74035. doi: 10.1002/adma.74035 (PMC13486210; doi:10.1002/adma.74035)
Supplement: Supplementary file 1 [file ADMA-38-e74035-s002.docx]

**Supporting Information**

**Reliable and reusable all-solid-state contact-type pre-lithiation** **platform for high-performance all-solid-state batteries**

Yunho Lee^a,b,+^, Yongjun Kwon^c,+^, Juhyeong Noh^a,b^, Seok Hun Kang^a,d^, Kyubeen Kang^e^, Junhyeok Choi^e^, Young-Gi Lee^a^, Jaecheol Choi^a,d,*^, Hyeong Min Jin^b,*^, Yong Min Lee^c,e,*^, Ju Young Kim^a,d,*^

^a^Smart Materials Research Section, Electronics and Telecommunications Research Institute (ETRI), 218 Gajeongno, Yuseong-gu, Daejeon 34129, Republic of Korea

^b^Department of Organic Materials Engineering, Chungnam National University, Daejeon, 34134, Republic of Korea

^c^Department of Chemical and Biomolecular Engineering, Yonsei University, 50 Yonsei-ro, Seodaemun-gu, Seoul 03722, Republic of Korea

^d^Department of Semiconductor and Advanced Device Engineering, University of Science and Technology (UST), Daejeon, 34113 Republic of Korea

^e^Department of Battery Engineering, Yonsei University, 50 Yonsei-ro, Seodaemun-gu, Seoul 03722, Republic of Korea

Corresponding authors

Jaecheol Choi: [jaecheol.choi@etri.re.kr](mailto:jaecheol.choi@etri.re.kr)

Hyeong Min Jin: [hyeongmin@cnu.ac.kr](mailto:hyeongmin@cnu.ac.kr)

Yong Min Lee: [yongmin@yonsei.ac.kr](mailto:yongmin@yonsei.ac.kr)

Ju Young Kim: [juyoung@etri.re.kr](mailto:juyoung@etri.re.kr)

^+^Yunho Lee and Yongjun Kwon contributed equally to this study.

**Experimental section**

*Preparation of materials*

Natural graphite and Li_6_PS_5_Cl (LPSCl) with average sizes (d_50_) of 16.8 and 3.0 μm were supplied by LG Chem. and Jeong Kwan Display Corp., respectively. Silicon particle with a diameter of <50 nm was purchased from Alfa Aesar. Lithium metal (300 μm thickness, Honjo Metal Corp.), anisole (Sigma-Aldrich), polyacrylic acid (PAA, Sigma-Aldrich) were used without further purification. The nitrile butadiene rubber (NBR, nitrile content ~32%) was supplied by LG Chem. As cathode materials, LiNi_0.6_Co_0.2_Mn_0.2_O_2_ (NCM622), LiCoO_2_ (LCO) were supplied by Umicore and L&F. Super P Li^®^ was purchased by Imerys. LiCl (Alfa Aesar) and InCl_3_ (Alfa Aesar) were used for Li_3_InCl_6_ (LIC) preparation. The precursor powders with a stoichiometric ratio of Li_3_InCl_6_ were milled using a planetary ball mill (Pulverisette 7PL, Fritsch GmbH) at 500 rpm for 10 h, and the resultant powders were thermally annealed at 260 °C for 5 h.

*Fabrication of mixed ionic–electronic conductor*

For the fabrication of the mixed ionic–electronic conductor (MIEC) pellet, LPSCl and carbon black were homogeneously mixed at a weight ratio of 95/5 wt%. Mixed powder of 130 mg was pelletized using a hydraulic uniaxial press at 550 MPa in polyether ether ketone mold with a diameter of 13 mm. The slurry-coated MIEC was prepared by mixing LPSCl, carbon black and a binder solution (5 wt% NBR in anisole). The slurries were homogeneously blended using a thinky mixer (ARM-310) and coated onto Ni foil through doctor blade casting, and dried at 130 °C for 30 min.

*Fabrication of all-solid-state electrode*

The diffusion-dependent electrodes (DDE) with different active material powders were prepared using a conventional slurry process. Active materials such as graphite, silicon and their blends were properly selected. The 5 wt% PAA binder dissolved in DI water were used and additional DI water was added to adjust the slurry viscosity. The slurries with active materials, PAA binder, and DI water were homogeneously blended using a thinky mixer and coated onto Ni foil using the doctor blade method. The electrode was heated at 100 °C for 1 hour and subsequently dried at 100 °C under vacuum overnight. The composite electrode was prepared by mixing graphite, Si nanoparticles, LPSCl, and a binder solution (5 wt% NBR in anisole). The slurries were homogeneously blended using a thinky mixer and coated onto Ni foil through doctor blade method, and dried at 100 °C for 1 h and subsequently dried at 100 °C under vacuum overnight. The NCM622/LPSCl cathode slurry was prepared by mixing active material, LPSCl, and NBR with a thinky mixer. The prepared slurries were subsequently coated onto Ni foil and dried in a convection oven at 130 °C for 30 min. For the composite electrode using LCO and LIC, LCO and LIC powders were homogeneously mixed at a weight ratio of 70/30 wt%, uniformly spread onto the LPSCl layer, and pressed under 550 MPa. All fabrication and pre-lithiation processes were performed in a dry room with a dew point of -60 °C.

*Fabrication of all-solid-state batteries*

For the fabrication of the all-solid-state batteries, the solid electrolyte layer was first formed using LPSCl powder. LPSCl powder of 200 mg was pelletized under 550 MPa in a polyether ether ketone mold with a diameter of 13 mm. The pristine DDE and pre-lithiated DDEs with a diameter of 13 mm were attached to one side of the LPSCl layer and pressed under 550 MPa. On the other side, lithium metal was attached to form a half cell. For the full cell configuration, the NCM622/LPSCl and LCO/LIC composite cathode were used.

*Characterization*

Scanning electron microscopy (SEM, SEC SNE-4500M, Hitachi S-4800) and energy dispersive X-ray spectroscopy (EDS, Bruker XFlash 640H Mini) were used for structural analysis and elemental mapping, respectively. X-ray diffraction (XRD, Malvern Panalytical Aeris) was performed to analyze crystalline structure. The Nyquist plot was obtained using a frequency response analyzer (Bio-logics SP-150) in a frequency range of 10^-1^ to 10^5^ Hz. The applied amplitude was 10 mV. Cyclic voltammetric (CV) analysis was performed at a scan rate of 0.1 mV/s in the same potential range applied for battery test. X-ray photoelectron spectroscopy (XPS) (Thermo Scientific, Nexsa G2) was conducted by mounting samples in an airtight vacuum-sealed holder in an argon glovebox before measurement. The charge/discharge properties of the all-solid-state batteries were investigated using a cycle tester (Toyo System) at 25 and 60 °C. The cut-off voltage was set as 0.01 ~ 2 V and 3 ~ 4.3 V depending on the cell configuration. Constant current/constant voltage mode (cutoff current: a fifth of the original current) was utilized for effective charge/discharge. All cells were tested under stack pressure of ~60 MPa.

*Formation of digital twin-driven electrode structures*
Virtual graphite DDE and MIEC structures were generated using the GrainGeo in GeoDict 2025 software. The DDE consisted of graphite (density = 2.22 g/cm^3^) and PVDF (1.78 g/cm^3^), and the MIEC consisted of LPSCl (1.88 g/cm^3^) and carbon black (2.00 g/cm^3^).^[1]^ Graphite and LPSCl particles were modeled as convex polyhedra, with D_50_ values of 16 μm and 2.59 μm, respectively. The virtual structures were constructed with dimensions of 50 μm $\times$ 50 μm $\times$ t (width $\times$ depth $\times$ thickness). A voxel resolution of 0.4 μm was selected to balance morphological fidelity and computational cost. The volume fractions of the DDE and MIEC components structures reproduced the experimentally derived target compositions with <5% deviation (Table S2).

Compression simulations of the DDE–MIEC structures were performed, applying stack pressures of 8, 24, 40, 56 and 72 MPa. All materials were assumed to be linearly elastic, and uniaxial compression was applied along the thickness (z-) direction at each stack pressure condition. To represent the confinement imposed by the polyether ether ketone mold, lateral deformation in the x- and y-directions was constrained, and symmetric boundary conditions were applied. The stress–strain relationship follows Hooke’s law:

$\sigma=C:\varepsilon$ (S1)

where $\sigma$ is the Cauchy stress tensor, $\varepsilon$ denotes the strain tensor, and $C$ is the fourth-order elasticity tensor defined by Young’s modulus ($E$) and Poisson’s ratio ($\nu$) of each material. The values of $E$ and $\nu$ used in the simulations are described in Table S3. When a small deformation is assumed, the mechanical equilibrium of the solid domain can be described by the following equation:

$\nabla\cdot\sigma\left( \varepsilon\right)=0$ (S2)

Also, the strain is linearly related to the displacement field $u$ as given by the kinematic equation:

$\varepsilon=\bar{\varepsilon}+\frac{1}{2}(\nabla u+\left( \nabla u \right)^{T})$ (S3)

where $\bar{\varepsilon}$ represents the macroscopic strain tensor and $u$ is the displacement vector.

*Electrochemical and mass transport simulations*

Electrochemical and mass transport simulations were performed in COMSOL Multiphysics 6.3 using the DDE–MIEC structures obtained from the compression simulations. The transport of Li^+^ and e^-^ within the MIEC was simulated using Ohm’s law:

$i_{l}=\sigma_{l}\nabla\phi_{l}$ (S4)

$i_{s}=\sigma_{s}\nabla\phi_{s}$ (S5)

where $\sigma_{l}$ and $\sigma_{s}$ are the ionic and electronic conductivities (S/cm^2^) of the MIEC, respectively, and $\phi_{l}$ and $\phi_{s}$ denote the electrochemical potentials in the LPSCl and carbon black domains. The interfacial charge-transfer current densities (mA/cm^2^) at the Li metal–MIEC and the MIEC–DDE interfaces were calculated using the Butler–Volmer equation:

$i_{loc}=i_{0}\left( \exp\left( \frac{\alpha_{a}F\eta}{RT} \right)-\exp\left( \frac{-\alpha_{c}F\eta}{RT} \right) \right)$ (S6)

where $i_{0}$ is the exchange current density (mA/cm^2^), $\alpha_{a}$ and $\alpha_{c}$ are the anodic and cathodic transfer coefficient, $\eta$ is the overpotential, $R$ is the ideal gas constant, $F$ is Faraday’s constant, and $T$ corresponds to 373.15 K (8 MPa) and 333.15K (56 MPa). Li^+^ intercalation occurs at the DDE–MIEC interface. Once intercalated, Li^+^ transport within the DDE proceeds solely by solid-state diffusion, as no volumetric generation or consumption of Li^+^ occurs in the electrode bulk. Therefore, Li^+^ concentration evolution inside the DDE is governed by diffusion without a source term:

$\frac{\partial c}{\partial t}+\nabla\cdot\left( -D\nabla c \right)=0$ (S7)

where $c$ represents the Li⁺ concentration (mol/m^3^) in the DDE and $D$ denotes the Li⁺ diffusion coefficient (cm²/s). The Li^+^ flux entering the DDE is supplied exclusively through interfacial charge-transfer reactions at the DDE–MIEC interface, which is imposed as a boundary condition:

$-n\cdot\left( -D\nabla C \right)=\frac{i}{F}$ (S8)

where $n$ is the unit normal vector at the interface, $i$ is the interfacial charge-transfer current density.

The key material parameters, including the lithium diffusion coefficient and exchange current density, were adopted from previous work, in which GITT analyses were performed on the graphite DDE at 60 °C.^[2]^ The corresponding parameters at 100 °C were calculated by considering temperature dependence using the Arrhenius equation. As a result, the average diffusion coefficients and exchange current densities at 60 °C and 100 °C were determined to be 2.3 × 10^-9^ cm^2^/s, 0.23 mA/cm^2^, and 3.2 × 10^-8^ cm^2^/s, 0.71 mA/cm^2^. The ionic and electronic conductivities used in the simulations were obtained from experimental measurements.

**Figure S1.** Charge-discharge voltage profiles at various rates from 0.1 C to 1.0 C-rate for (a) graphite diffusion-dependent electrode (Gr DDE) and (b) pre-cycled Gr DDE. Schematic illustration of the cell structure for (c) the pristine Gr DDE and (d) pre-cycled Gr DDE. SEI formation on the surface of graphite during the formation step influences lithium-ion diffusion for the lithiation of the electrode. Because the electric field is screened by abundant electrons of graphite, the driving force for lithium-ion conduction through the SEI is absent. Therefore, the SEI formed on graphite particles within the electrode can serve as a physical diffusion barrier, impeding interparticle transport and thereby hindering lithiation throughout the electrode. This highlights the critical importance of interfacial properties in all-solid-state battery systems.

 **Figure S2.** Nyquist plot result at different LPSCl/carbon black weight ratios of (a) 97/3 wt%, (b) 95/5 wt%, and (c) 93/7 wt% measured using an ion-blocking symmetric cell configuration.

**Figure S3.** Nyquist plots of the LPSCl/carbon black (95/5 wt%) composite at different temperatures of (a) 40 °C, (b) 60 °C, (c) 80 °C, and (d) 100 °C measured using an electron-blocking symmetric cell configuration.

**Figure S4.** (a) Nyquist plots and (b) ionic conductivity of the LPSCl pellet at different temperatures.

**Figure S5.** Images of the separability of the target electrode from the MIEC under applied stack pressures of (a) 8 MPa, (b) 24 MPa, and (c) 56 MPa.

**Figure S6.** Charge-discharge voltage profiles of half cells using Gr/n-Si DDE pre-lithiated at 60 °C for 30 minutes under stack pressures of (a) pristine, (b) 8 MPa, (c) 24 MPa, and (d) 56 MPa.

**Figure S7.** Cycling voltammetry of (a) pristine DDE and (b) pre-lithiated DDE at 60 °C for 30 minutes under 56 MPa (Scan rate: 0.1 mV/s, voltage range: 0.0 V ~ 2.0 V vs. Li/Li^+^)..

**Figure S8.** Nyquist plot results of the MICE at different pressures using an ion-blocking symmetric cell configuration. Schematic illustration of ion blocking symmetric cells using the MIEC and DDEs, and the corresponding equivalent circuit. In the low-frequency region, ions in the MIEC are blocked by the electrodes. As a result, the current flow is governed exclusively by electronic conduction. Subsequently, by subtracting the resistances of the electrode and the MIEC from the total low-frequency resistance, the interfacial resistance between the electrode and the MIEC can be calculated.

**Table S1.** Resistance values of each component.

**Figure S9.** Cycling voltammetry of the DDE half-cells under stack pressure of (a) 550 MPa, (b) 56 MPa, and (c) 8 MPa (Scan rate: 0.1 mV/s, voltage range: 0.0 V ~ 2.0 V vs. Li/Li^+^).

**Figure S10.** Charge-discharge voltage profiles of the (a) pristine DDE and (b-e) PL-series half cells performed at 60 ℃ under a stack pressure of 56 MPa.

**Figure S11.** Linear relationship between pre-lithiated capacity and contact duration for pre-lithiation (a) under 56 MPa at 60 ℃ and (b) 8 MPa at 100 ℃.

**Figure S12.** Optical images of the (a) pristine DDE surface and PL-series DDE surfaces conducted at 60 ℃ under a stack pressure of 56 MPa for (b) 30 min and (c) 60 min.

**Figure S13.** Optical images of the (a) pristine Gr DDE surface and PL-series Gr DDE surfaces conducted at 60 ℃ under a stack pressure of 56 MPa for (b) 30 min and (c) 60 min.

**Figure S14.** Cross-sectional optical images of the Gr DDE demonstrating the uniform elemental distribution of lithium across the electrode thickness.

**Figure S15.** Optical images of (a) pristine n-Si DDE surface and (b) PL-30 n-Si DDE surfaces conducted at 60 ℃ under a stack pressure of 56 MPa. Charge-discharge voltage profiles of (c) pristine n-Si DDE and (d) PL-30 n-Si DDE half cells performed at 60 ℃ under a stack pressure of 56 MPa.

**Figure S16.** (a) Time-dependent monitoring of the OCV of the PL-60 graphite DDE at 25 °C. Operando OM images showing surface change of the PL-60 graphite DDE at 25 ℃; (b) Pristine, (c) after 4 hr, (d) 8 hr, (e) 12 hr, (f) 16 hr, and (g) 20 hr.

**Figure S17.** X-ray diffraction results of (a) graphite and n-Si and (b-c) as a function of pre-lithiation time for Gr/n-Si DDEs.

**Figure S18.** Cross-sectional SEM images of (a) pristine, (b) PL-30 and (c) PL-60 DDEs.

**Figure S19.** (a) Comparison of ICE of pre-lithiated (PL-45) full cells with different cathodes and the corresponding cathode half cells. First-cycle voltage profiles of (b) LCO and (c) NCM622 half cells and full cells paired with PL-45.

**Figure S20.** C 1s, Si 2p and Li 1s XPS spectra of the (a) pristine, (b) PL-30 and (c) PL-60 DDEs.

**Figure S21.** (a) ICE and OCV of Gr/n-Si DDE full-cells pre-lithiated at 60 ℃ under 8 MPa as a function of pre-lithiation times. Charge-discharge voltage profiles of the (b-e) PL-series full cells.

**Figure S22.** Cycling voltammetry of the DDE half-cells under stack pressure of 8 MPa at 100 ℃ (Scan rate: 0.1 mV/s, voltage range: 0.0 V ~ 2.0 V vs. Li/Li^+^).

**Figure S23.** Schematic illustrations depicting the effect of contact-area control during pre-lithiation on interfacial properties.

**Figure S24.** C 1s, Si 2p and Li 1s depth-XPS spectra of the (a) PL-56-60 and (b) PL-100-8 DDEs

**Figure S25.** Charge-discharge voltage profiles of (a) the pristine DDE and (b-e) PL-series half cells pre-lithiated under a stack pressure of 8 MPa at 100 ℃.

**Figure S26.** Digital twin structures of graphite DDE and MIEC.

**Table S2.** Target and model volume fractions of DDE and MIEC components and the corresponding deviations.

**Table S3.** Mechanical properties of the materials used in the compression simulations.

**Figure S27.** von Mises stresses exceeding the critical yield stress of LPSCl (20-60 MPa) under stack pressures of 8 and 56 MPa.

**Figure S28.** Average state of lithiation as a function of z-coordinate at 30 and 60 min under different pre-lithiation conditions, quantitatively extracted from the digital twin simulation shown in Figure 4d, e.

**Figure S29.** (a) ICE and OCV of Gr/n-Si DDEs full-cells pre-lithiated at 100 ℃ under 8 MPa as a function of pre-lithiation times. Charge-discharge voltage profiles of (b) pristine DDE and (c-e) PL-series full cells performed at 100 ℃ under a stack pressure of 8 MPa.

**Table S4.** Comparison of the ICE, initial discharge capacity at different C-rates (0.1C and 0.3C), N/P ratio, and the evolution of discharge capacity and capacity retention at the 100th, 200th, and 300th cycles for pristine and pre-lithiated electrodes at 25 °C.

**Figure S30.** (a) Charge-discharge voltage profiles of NCM622 half-cell at 25 ℃. Charge-discharge voltage profiles of (b) pristine DDE and PL-series full cells performed at 100 ℃ under 8 MPa for (c) PL-20, and (d) PL-30 at 25 ℃.

**Table S5.** Comparison of the ICE, initial discharge capacity at different C-rates (0.1C and 0.3C), N/P ratio, and the evolution of discharge capacity and capacity retention at the 100th, 200th, and 300th cycles for pristine and pre-lithiated electrodes at 25 °C.

**Figure S31.** (a) Rate performance of the all-solid-state pristine DDE and the PL-8-45 at 60 ℃. Charge-discharge voltage profiles at various rates from 0.1C to 1.0 C-rate for all-solid-state (b) pristine DDE and (c) PL-8-45. (d) Rate performance of the all-solid-state pristine DDE and the PL-8-20 at 25 ℃. Charge-discharge voltage profiles at various rates from 0.1C to 0.7 C-rate for all-solid-state (e) pristine DDE and (f) PL-8-20.

**Figure S32.** (a) First-cycle voltage profiles of the PL-8-45 and PL-8-60. (b) Capacity retention performance and Coulombic efficiency of the pristine DDE and PL-series full-cells with different pre-lithiation time (45 min and 60 min).

**Figure S33.** First-cycle voltage profiles of (a) five PL-30 and (b) five PL-45 electrodes.

**Table S6.** The initial OCV, charge–discharge capacities, and ICEs of five PL-30 and five PL-45 electrodes.

**Figure S34.** (a) XRD patterns, (b) Nyquist plots, and (c) SEM images of the MIEC before and after multiple pre-lithiation (up to 5 cycles).

**Figure S35.** (a) Photograph and (b) Nyquist plots of the slurry-coated MIEC. (c) Top-view SEM images and (d) corresponding EDS images of the slurry-coated MIEC. (e) Conceptual schematic illustration of the all-solid-state MIEC-based pre-lithiation.

**Table S7.** Summary of reported pre-lithiation strategies, including methods, process conditions, and electrochemical performance.

**References**

[1]	S. Kim, H. Lee, J. Lim, et al., "Digital Twin Battery Modeling and Simulations: A New Analysis and Design Tool for Rechargeable Batteries," ACS Energy Letters 9 (2024): 5225–5239.

[2]	J. Y. Kim, J. Choi, J. Choi, et al., "Mechanical Stress-Tolerant Diffusion-Dependent Electrode with Well-dispersed Silicon Particles for High-Performance All-Solid-State Batteries," Carbon Energy 7 (2025): e70046.

[3]	A. Verma, A. Singh, A. Colclasure, et al., "On the impact of mechanics on electrochemistry of lithium-ion battery anodes," JOM 76 (2024): 1171–1179.

[4]	L. S. De Vasconcelos, N. Sharma, R. Xu, et al., "In-situ nanoindentation measurement of local mechanical behavior of a Li-ion battery cathode in liquid electrolyte," Experimental Mechanics 59 (2019): 337–347.

[5]	K. Hikima, M. Totani, S. Obokata, et al., "Mechanical properties of sulfide-type solid electrolytes analyzed by indentation methods," ACS Applied Energy Materials 5 (2022): 2349–2355. [6]	W. Yang, S. Mao, J. Yang, et al., "Large-deformation and high-strength amorphous porous carbon nanospheres," Scientific Reports 6 (2016): 24187.
